# Supplementary material for: Repurposed drugs and their combinations prevent morbidity-inducing dermonecrosis caused by diverse cytotoxic snake venoms
Source: Nat Commun. 2023 Dec 14;14:7812. doi: 10.1038/s41467-023-43510-w (PMC10721902; doi:10.1038/s41467-023-43510-w)
Supplement: Supplementary file 2 — Reporting Summary [file 41467_2023_43510_MOESM2_ESM.pdf]

nature portfolio

Corresponding author(s): Prof. Nicholas Casewell

Last updated by author(s): Oct 3, 2023

Reporting Summary

Nature Portfolio wishes to improve the reproducibility of the work that we publish. This form provides structure for consistency and transparency in reporting. For further information on Nature Portfolio policies, see our Editorial Policies and the Editorial Policy Checklist.

Statistics

For all statistical analyses, confirm that the following items are present in the figure legend, table legend, main text, or Methods section.

n/a	Confirmed	
☐	☒	The exact sample size (*n*) for each experimental group/condition, given as a discrete number and unit of measurement
☐	☒	A statement on whether measurements were taken from distinct samples or whether the same sample was measured repeatedly
☐	☒	The statistical test(s) used AND whether they are one- or two-sided<br>*Only common tests should be described solely by name; describe more complex techniques in the Methods section.*
☒	☐	A description of all covariates tested
☒	☐	A description of any assumptions or corrections, such as tests of normality and adjustment for multiple comparisons
☐	☒	A full description of the statistical parameters including central tendency (e.g. means) or other basic estimates (e.g. regression coefficient) AND variation (e.g. standard deviation) or associated estimates of uncertainty (e.g. confidence intervals)
☐	☒	For null hypothesis testing, the test statistic (e.g. F, t, r) with confidence intervals, effect sizes, degrees of freedom and P value noted<br>*Give P values as exact values whenever suitable.*
☒	☐	For Bayesian analysis, information on the choice of priors and Markov chain Monte Carlo settings
☒	☐	For hierarchical and complex designs, identification of the appropriate level for tests and full reporting of outcomes
☒	☐	Estimates of effect sizes (e.g. Cohen's *d*, Pearson's *r*), indicating how they were calculated

Our web collection on statistics for biologists contains articles on many of the points above.

Software and code

Policy information about availability of computer code

Data collection	FLUOstar Omega microplate reader software v5.70 (BMG LABTECH), SoftMax Pro v7.1 (Molecular Devices), Revolve-M30 microscope software v4.0.1 (Echo)
Data analysis	Excel v2204 (Microsoft), Prism v9.3.1 (GraphPad)

For manuscripts utilizing custom algorithms or software that are central to the research but not yet described in published literature, software must be made available to editors and reviewers. We strongly encourage code deposition in a community repository (e.g. GitHub). See the Nature Portfolio guidelines for submitting code & software for further information.

Data

Policy information about availability of data

All manuscripts must include a data availability statement. This statement should provide the following information, where applicable:

- Accession codes, unique identifiers, or web links for publicly available datasets
- A description of any restrictions on data availability
- For clinical datasets or third party data, please ensure that the statement adheres to our policy

There are no restrictions on data availability. Source data are provided with this paper. The H&E-stained dermal cross-sections of the animals' injection sites used for histopathological analysis have been deposited in the Figshare database under accession code: 10.6084/m9.figshare.19706761.v1

Research involving human participants, their data, or biological material

Policy information about studies with human participants or human data. See also policy information about sex, gender (identity/presentation), and sexual orientation and race, ethnicity and racism.

Reporting on sex and gender	N/A (no human data used)
Reporting on race, ethnicity, or other socially relevant groupings	N/A
Population characteristics	N/A
Recruitment	N/A
Ethics oversight	N/A

Note that full information on the approval of the study protocol must also be provided in the manuscript.

Field-specific reporting

Please select the one below that is the best fit for your research. If you are not sure, read the appropriate sections before making your selection.

☒ Life sciences ☐ Behavioural & social sciences ☐ Ecological, evolutionary & environmental sciences

For a reference copy of the document with all sections, see nature.com/documents/nr-reporting-summary-flat.pdf

Life sciences study design

All studies must disclose on these points even when the disclosure is negative.

Sample size	In animal trials, sample sizes (5 mice per treatment group) were rationally selected based on our group's prior hemorrhage research (see Albulescu et al. 2020 Science Translational Medicine) and to enable meaningful comparisons between different small molecule drug therapies. As we had never previously completed in vivo snake venom-induced dermonecrosis work using small molecule drug inhibitors we had no available estimates of their potential effectiveness (or ineffectiveness), and therefore appropriate power calculations could not be completed to pre-determine sample size, hence our decision to complete our experiments with an n of 5 to mimic previous related studies.<br><br>For cellular experiments, a minimum experimental replicate sample size 'n' of 3 was used to allow for sufficient statistical comparisons of data.
Data exclusions	No data was excluded.
Replication	For cellular trials, each treatment within each experiment had a minimum of three technical replicates. Then, each independent experiment was repeated a minimum of three times to achieve a minimum 'n' value of 3. Not all attempts were successful, as initial trials used concentrations of venom that were too low to achieve 100% cell death at the highest concentrations used, meaning these early trials did not produce usable dose-response curve nor IC50/EC50 data; therefore, replicates using higher venom concentrations were then completed to achieve the appropriate 'n' value for each treatment.<br><br>For mice, each separate animal was considered a replicate 'n'. Not all replicates were successful, due to issues such as mis-dosing or external lesions reaching our pre-defined humane endpoint maximum size (10 mm) prior to the 72h dermonecrosis endpoint, resulting in early animal euthanasia. This explains why some of our in vivo data only had a final 'n' of 4 rather than the originally planned 5.
Randomization	Animal and cell assignation to experimental groups was randomized.
Blinding	Experimenters were blinded to drug- or vehicle-treatments during animal studies. Experimenters were not blinded during cellular experiments, since all cell work was completed by a single individual.

Reporting for specific materials, systems and methods

We require information from authors about some types of materials, experimental systems and methods used in many studies. Here, indicate whether each material, system or method listed is relevant to your study. If you are not sure if a list item applies to your research, read the appropriate section before selecting a response.

Materials & experimental systems

n/a	Involved in the study
☒	☐ Antibodies
☐	☒ Eukaryotic cell lines
☒	☐ Palaeontology and archaeology
☐	☒ Animals and other organisms
☒	☐ Clinical data
☒	☐ Dual use research of concern
☒	☐ Plants

Methods

n/a	Involved in the study
☒	☐ ChIP-seq
☒	☐ Flow cytometry
☒	☐ MRI-based neuroimaging

Eukaryotic cell lines

Policy information about cell lines and Sex and Gender in Research

Cell line source(s)	HaCaT immortalised human keratinocytes were purchased from Caltag Medsystems (Buckingham, UK), who distribute the cells from the company AddexBio (San Diego, USA).
Authentication	The HaCaT cells were verified by AddexBio using STR profiling.
Mycoplasma contamination	Cells were verified mycoplasma free from AddexBio and Caltag before shipment.
Commonly misidentified lines (See ICLAC register)	HaCaT cells are not on the ICLAC register of commonly misidentified cell lines.

Animals and other research organisms

Policy information about studies involving animals; ARRIVE guidelines recommended for reporting animal research, and Sex and Gender in Research

Laboratory animals	Liverpool, UK: 18-20 g [4-5 weeks old], male, CD-1 mice purchased from Charles River, UK.<br>San Jose, Costa Rica: 18-20 g [4-5 weeks old], mixed sex, CD-1 mice obtained from Instituto Clodomiro Picado (ICP, Costa Rica).
Wild animals	No wild animals were used.
Reporting on sex	Sex was not considered in the design or analysis of this study, as the goal was not to determine the effect of sex on drug or venom effects, but simply whether the direct inhibition of these toxins with drug inhibitors could reduce the necrosis caused by the venoms in vivo. However, across the whole study, both sexes of animals were used.
Field-collected samples	Study did not involved field-collected samples.
Ethics oversight	All animal experiments done in Liverpool were conducted using protocols approved by the Animal Welfare and Ethical Review Boards of the Liverpool School of Tropical Medicine and the University of Liverpool and were performed in pathogen-free conditions under licensed approval (PPL #P58464F90) of the UK Home Office and in accordance with the Animal [Scientific Procedures] Act 1986 and institutional guidance on animal care. All animal experiments done in Costa Rica were conducted using protocols approved by the Institutional Committee for the Care and Use of Laboratory Animals (CICUA) of the University of Costa Rica (approval number CICUA 82-08).

Note that full information on the approval of the study protocol must also be provided in the manuscript.
